# Transition of care at discharge from the Intensive Care Unit: a scoping review*

**DOI:** 10.1590/1518-8345.4008.3325

**Published:** 2020-07-15

**Authors:** Michele Elisa Weschenfelder Hervé, Paula Buchs Zucatti, Maria Alice Dias Da Silva Lima

**Affiliations:** 1 Universidade Federal do Rio Grande do Sul, Porto Alegre, RS, Brazil.; 2 Hospital de Clínicas de Porto Alegre, Centro de Terapia Intensiva, Porto Alegre, RS, Brazil.; 3 Hospital Conceição, Unidade de Terapia Intensiva, Porto Alegre, RS, Brazil.

**Keywords:** Critical Care, Intensive Care Units, Patient Transfer, Continuity of Patient Care, Patient Discharge, Patient Handoff, Cuidados Críticos, Unidades de Terapia Intensiva, Transferência de Pacientes, Continuidade da Assistência ao Paciente, Alta do Paciente, Transferência da Responsabilidade pelo Paciente, Cuidados Críticos, Unidades de Cuidados Intensivos, Transferencia de Pacientes, Continuidad de la Atención al Paciente, Alta del Paciente, Pase de Guardia

## Abstract

**Objective:**

to map the available evidence on the components of the transition of care, practices, strategies, and tools used in the discharge from the Intensive Care Unit (ICU) to the Inpatient Unit (IU) and its impact on the outcomes of adult patients.

**Method:**

a scoping review using search strategies in six relevant health databases.

**Results:**

37 articles were included, in which 30 practices, strategies or tools were identified for organizing and executing the transfer process, with positive or negative impacts, related to factors intrinsic to the Intensive Care Unit and the Inpatient Unit and cross-sectional factors regarding the staff. The analysis of hospital readmission and mortality outcomes was prevalent in the included studies, in which trends and potential protective actions for a successful care transition are found; however, they still lack more robust evidence and consensus in the literature.

**Conclusion:**

transition of care components and practices were identified, in addition to factors intrinsic to the patient, associated with worse outcomes after discharge from the Intensive Care Unit. Discharges at night or on weekends were associated with increased rates of readmission and mortality; however, the association of other practices with the patient’s outcome is still inconclusive.

## Abstract (pt)

**Objetivo:**

mapear as evidências disponíveis sobre os componentes da transição do cuidado, as práticas, estratégias e ferramentas utilizadas na alta da Unidade de Terapia Intensiva para a Unidade de Internação e seu impacto nos desfechos de pacientes adultos.

**Método:**

revisão de escopo mediante estratégias de busca em seis bases de dados relevantes na área da saúde.

**Resultados:**

foram incluídos 37 artigos, nos quais foram identificadas 30 práticas, estratégias ou ferramentas para organização e execução do processo de transferência, com impactos positivos ou negativos, relacionadas a fatores intrínsecos à Unidade de Terapia Intensiva, à Unidade de Internação e transversais às equipes envolvidas. A análise dos desfechos ‘readmissão’ e ‘mortalidade’ foi prevalente nos estudos incluídos, nos quais se encontram tendências e potenciais ações protetoras para uma transição do cuidado bem-sucedida, contudo, ainda carecem de evidências mais robustas e consenso na literatura.

**Conclusão:**

foram identificados componentes e práticas de transição do cuidado, além de fatores intrínsecos ao paciente, associados a piores desfechos após a alta da Unidade de Terapia Intensiva. A alta noturna ou em finais-de-semana mostrou associação com aumento nas taxas de readmissão e mortalidade, porém, a associação das demais práticas com o desfecho do paciente ainda é inconclusiva.

## Abstract (es)

**Objetivo:**

mapear la evidencia disponible sobre los componentes de la transición del cuidado, las prácticas, las estrategias y las herramientas que se utilizan al momento del alta de la Unidad de Cuidados Intensivos a la Unidad de Internación y su efecto sobre los resultados de pacientes adultos.

**Método:**

una revisión sistemática exploratoria en la que se emplean estrategias de búsqueda en seis bases de datos sobre salud relevantes.

**Resultados:**

se incluyeron 37 artículos, en los cuales se identificaron 30 prácticas, estrategias o herramientas para organizar y ejecutar el proceso de transferencia, con efectos positivos o negativos, relacionados con factores intrínsecos a la Unidad de Cuidados Intensivos y a la Unidad de Internación, además de factores transversales relacionados con el personal. El análisis de los resultados de reinternación y mortalidad fue prevalente en los estudios incluidos, en los cuales pueden encontrarse tendencias y acciones potencialmente protectoras para una transición de cuidados exitosa; sin embargo, siguen careciendo de evidencias y consenso más sólidos en la literatura.

**Conclusión:**

se identificaron componentes y las prácticas de la transición del cuidado, además de factores intrínsecos al paciente, asociados con peores resultados después del alta de la Unidad de Cuidados Intensivos. Las altas nocturnas o durante los fines de semana se asociaron con índices más elevados de reinternaciones y mortalidad; sin embargo, la asociación de otras prácticas con el resultado del paciente sigue sin ser concluyente.

## Introduction

Transition of care refers to a set of actions aimed at the coordination and continuity of care in the transfer of patients between different locations in the health system, or between different levels of care within the same institution^(^ 1 ^)^. The quality of transition of care is used as one of the components for evaluating the performance of hospitals by the World Health Organization (WHO) and by the Joint Commission International (JCI), and is related to the International Patient Safety Goal 2 - Communication^(^ 1 ^-^ 2 ^)^.

It is a complex process involving several elements and stages, such as effective communication, patient and family guidance, responsibility of each of the parties involved, discharge planning, and knowledge about the resources and structure of the destination scenario, among others^(^ 1 ^,^ 3 ^)^. Therefore, the process is extremely vulnerable to the loss of critical information and to failures in the continuity of care. An inadequate transition of care can lead to serious adverse events, omission of care, duplication of care, delays in treatment, receiving inadequate treatment, increased morbidity and mortality, in addition to dissatisfaction of the patient, family and professionals, the inadequate use of health services and increased costs^(^ 2 ^)^.

The transition of care from the Intensive Care Unit (ICU) to the Inpatient Unit (IU) is related to an even higher risk due to a combination of factors such as the severity of the patients, multiple comorbidities and complexity of the care, change from an environment with many monitoring resources to an environment with fewer resources, number and complexity of the professionals involved (multidisciplinary and inter-specialties), lack of transition programs or lack of standardization of the discharge process, in addition to frequent oral and written miscommunication between the staff and between professionals and the patient/family^(^ 4 ^-^ 7 ^)^. Despite the growing knowledge on the subject, the quality of transition practices is still very varied, with deficits in planning, coordination of care and exchange of information between ICU and IU health professionals^(^ 7 ^)^.

The occurrence of adverse events after discharge from the ICU is related to events with medication, falls and nosocomial infection, clinical deterioration, cardiorespiratory arrest, readmission and death^(^ 8 ^-^ 11 ^)^. However, studies that assess the occurrence of other outcomes are still scarce; and most focus only on readmission and death rates^(^ 6 ^,^ 12 ^-^ 14 ^)^. The association between the occurrence of such events and the poor quality of the transition of care is demonstrated in some studies; however, the literature is still controversial on the topic^(^ 4 ^,^ 13 ^-^ 14 ^)^.

The adoption of standardized and precise guidelines is important to determine the ideal time for discharge, as well as to predict patients at greatest risk of suffering adverse events after the transfer. However, risk factors and discharge criteria are not clearly defined^(^ 8 ^,^ 10 ^-^ 11 ^,^ 15 ^)^. Although guidelines and transition programs are considered effective management tools to reduce length of stay and improve use of resource, few institutions have a policy regarding transition of care or written guidelines for the discharge process from the ICU^(^ 4 ^,^ 15 ^)^.

A scoping review conducted in 2015^(^ 4 ^)^ about patients discharged from the ICU to inpatient units analyzed studies published until 2013, without age or clinical profile restrictions, including adult, pediatric, and neonatal patients. The results indicated components or stages for an ICU discharge strategy, such as institutional guidelines to standardize the processes regarding transition of care, risk stratification of patients, training of professionals and adoption of a discharge plan. In addition, determining the best day and time for discharge, reducing transfer delays, oral communication between providers, a verification checklist before transfer, patient follow-up, and evaluation of post-discharge outcomes are also mentioned as important elements^(^ 4 ^)^. The gap in the review^(^ 4 ^)^ is the need to assess the elements identified, adapted to local needs and contexts before widespread implementation^(^ 4 ^)^. The association between different discharge practices and patient outcomes was also not assessed.

Thus, this study intends to map the available evidence on the components of the transition of care, the practices, strategies and tools used in the discharge of patients from the ICU to the IU and the impact on the outcomes of adult patients.

## Method

The knowledge synthesis method adopted was the scoping review^(^ 16 ^)^. The following phases were developed according to the methodology proposed by the Joanna Briggs Institute^(^ 17 ^)^: definition and alignment of research objectives and questions; establishing inclusion criteria according to the objectives and questions; elaboration and planning of the study search and selection strategy; identification of relevant studies; selection of studies; data extraction; data mapping; and summarizing the results.

This investigation was guided by the following questions: What are the components of the transition of patients from ICU to IU according to the literature? What practices, strategies, and tools are associated with improving the quality of discharge from the ICU to the IU? What is its impact on patients’ outcomes after the transfer?

The inclusion criteria for the selection included the following: primary studies carried out with adult patients (18 years old or older); published in English, Spanish or Portuguese; and in the period from January 1^st^, 2014 to December 31^st^, 2018. The delimitation of this period is justified because there is already in the literature a scoping review on the topic that included studies until 2013^(^ 4 ^)^.

Duplicate articles, those that did not answer at least one of the research questions, review studies, books, letters to the editor, abstracts published in annals and studies about patients transferred from the ICU for psychiatric, obstetric or palliative care were excluded, due to the particularities in the care of these patients and because they are frequently transferred to specialized inpatient units, limiting the comparison of results.

The search strategy consisted of three stages: i) Initial research in the PubMed and Cumulative Index to Nursing and Allied Health Literature (CINAHL) databases using the descriptors found in the Medical Subject Headings (MeSH): *critical care, intensive care unit, patient transfer, continuity of patient care, patient handoff, patient handover, patient care team, communication, patient discharge, patient readmission*, followed by an analysis of the keywords contained in the title, summary and descriptors of the articles, identifying uncontrolled descriptors: *care transitions, discharge practices* and *discharge planning*; ii) Second search using all the descriptors identified in the included databases -PubMed, CINAHL, Latin American and Caribbean Health Sciences Literature (*Literatura Latino-Americana e do Caribe em Ciências da Saúde*, LILACS), *Web of Science*, Scopus, and Embase. The boolean operators OR and AND were used, as shown in Figure 1; iii) A search was carried out in the references of the included articles in order to track additional studies not identified by the search strategies. The study selection process, as well as the last search, took place in April and May 2019.

**Figure 1:** Database search strategy using boolean operators. Porto Alegre, RS, Brazil, 2019

Database	Strategy	Limiters
PubMed	Search (“Intensive Care Units”[Mesh] OR “critical care”[tw] OR “intensive care”[tw]) AND (“Continuity of Patient Care”[Mesh] OR “Continuity of Patient Care”[tw] OR (“Patient Discharge*”[tw] AND “patient readmission*”[tw]) OR “discharge practice*”[tw] OR “discharge planning”[tw] OR “Patient Handoff*”[tw] OR “Patient Transfer*”[tw] OR “Patient Handover*”[tw] OR (“patient care team*”[tw] AND “communicat*”[tw]) OR “care transition*”[tw]) AND (adult*[tw] OR “Adult”[Mesh] OR aged[tw])	Journal Article; published in the last 5 years; Humans; English; Portuguese; Spanish
CINAHL	TX ((“Intensive Care Units” OR “critical care” OR “intensive care”) AND (“Continuity of Patient Care” OR (“Patient Discharge*” AND “patient readmission*”) OR “discharge practice*” OR “discharge planning” OR “Patient Handoff*” OR “Patient Transfer*” OR “Patient Handover*” OR (“patient care team*” AND “communicat*”) OR “care transition*”)) AND AG (adult* OR aged)	Publication date: 20130101-20181231; Language: English, Portuguese, Spanish
LILACS	(“Intensive Care Units” OR “critical care” OR “intensive care”) AND (“Continuity of Patient Care” OR (“Patient Discharge*” AND “patient readmission*”) OR “discharge practice*” OR “discharge planning” OR “Patient Handoff*” OR “Patient Transfer*” OR “Patient Handover*” OR (“patient care team*” AND “communicat*”) OR “care transition*”) [Palavras]	2013 OR 2014 OR 2015 OR 2016 OR 2017 OR 2018 [Country, year of publication]
Web of Science	Topic: ((“Intensive Care Units” OR “critical care” OR “intensive care”) AND (“Continuity of Patient Care” OR (“Patient Discharge*” AND “patient readmission*”) OR “discharge practice*” OR “discharge planning” OR “Patient Handoff*” OR “Patient Transfer*” OR “Patient Handover*” OR (“patient care team*” AND “communicat*”) OR “care transition*”) AND (adult* OR aged))	Years of the publication: (2018 OR 2017 OR 2016 OR 2015 OR 2014 OR 2013) AND Languages: (ENGLISH OR PORTUGUESE OR SPANISH)
Scopus	TITLE-ABS-KEY (“Intensive Care Units” OR “critical care” OR “intensive care”) AND TITLE-ABS-KEY (“Continuity of Patient Care” OR (“Patient Discharge*” AND “patient readmission*”) OR “discharge practice*” OR “discharge planning” OR “Patient Handoff*” OR “Patient Transfer*” OR “Patient Handover*” OR (“patient care team*” AND “communicat*”) OR “care transition*”) AND TITLE-ABS-KEY (adult* OR aged)	(LIMIT-TO (PUBYEAR, 2018) OR LIMIT-TO (PUBYEAR, 2017) OR LIMIT-TO (PUBYEAR, 2016) OR LIMIT-TO (PUBYEAR, 2015) OR LIMIT-TO (PUBYEAR, 2014) OR LIMIT-TO (PUBYEAR, 2013)) AND (LIMIT-TO (LANGUAGE, “English”) OR LIMIT-TO (LANGUAGE, “Spanish”) OR LIMIT-TO (LANGUAGE, “Portuguese”))
Embase	All fields (‘intensive care’ OR ‘intensive care unit’) AND (‘patient transfer’ OR (‘patient care’ AND ‘interpersonal communication’) OR ‘clinical handover’ OR ‘care transition’) AND ([adult]/lim OR [aged]/lim OR [middle aged]/lim OR [very elderly]/lim OR [young adult]/lim)	(2013:py OR 2014:py OR 2015:py OR 2016:py OR 2017:py OR 2018:py) AND ([english]/lim OR [portuguese]/lim OR [spanish]/lim)

The selected references were sent to the Mendeley^®^ bibliographic managing software. Two researchers worked independently to select the studies by title, abstract, and full text. The two reviewers evaluated the full versions of the text of the selected articles, considering the inclusion and exclusion criteria, resulting in the final study sample. In each phase, a consensus was reached between the reviewers through discussion.

The researchers prepared a data extraction form to record the characteristics of the included studies and the main information relevant to the research, containing the following sections: author(s), title, journal, country, year, volume, number, objective(s), population, sample size, method, how the results were measured, main findings, and study category. The impact of the transition of care practices, strategies or tools was interpreted as positive or negative, through the researchers’ consensus after extracting the results independently, according to the effect on the quality of the transition of care, its implementation, and the conclusion of its stages and/or according to the association with the patients’ outcomes.

## Results

**Figure 2:**
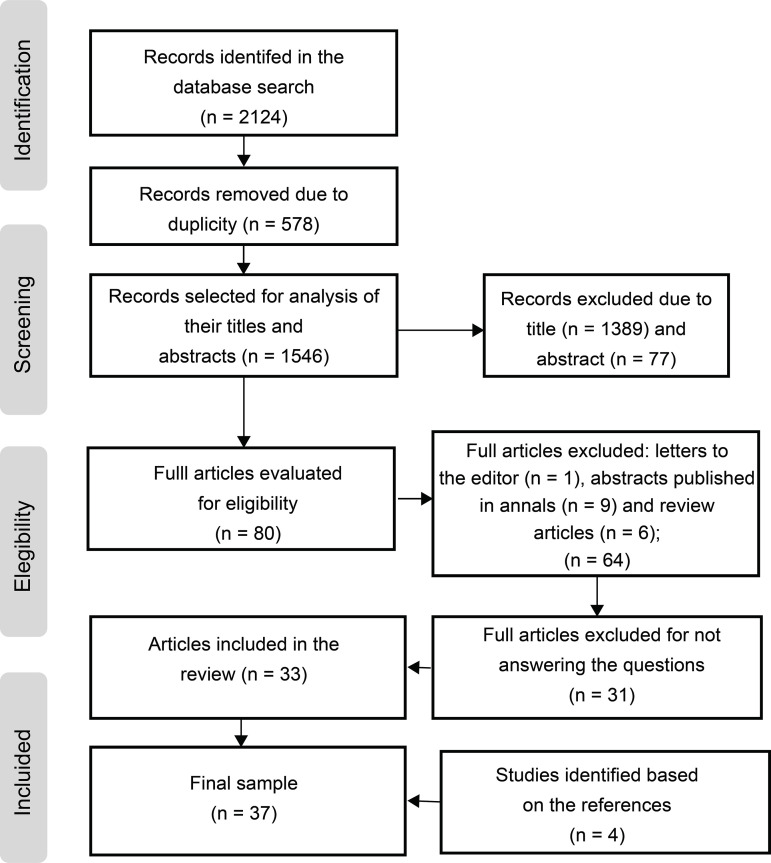
Flowchart of the study selection process adapted from *Preferred Reporting Items for Systematic Review and Meta-Analyses* (PRISMA)^(^ 18 ^)^. Porto Alegre, RS, Brazil, 2019

The search in the databases identified 2,124 potentially eligible studies and another four articles were selected from the references, 37 remaining in the final sample, as shown in Figure 2.

The characteristics of the articles are summarized in Table 1. The research studies were carried out mainly in the United States, Canada, and Australia, and the methodology was quite varied, with a bigger number of qualitative, cohort, and quasi-experimental studies.

**Table 1:** Distribution of the included studies according to country, type of study, and year of publication. Porto Alegre, RS, Brazil, 2019

Characteristics	n*	%^†^
**Country**		
United States	8	21.6
Canada	6	16.2
Australia	4	10.8
Australia/New Zealand	3	8.1
Sweden	3	8.1
United Kingdom	3	8.1
Netherlands	2	5.4
South Korea	2	5.4
Brazil	1	2.7
Argentina	1	2.7
Uruguay	1	2.7
Norway	1	2.7
Belgium	1	2.7
United States, Canada, and United Kingdom	1	2.7
Type of study		
Quasi-experimental	7	18.9
Prospective cohort	7	18.9
Qualitative	7	18.9
Descriptive	6	16.2
Retrospective cohort	5	13.5
Mixed (qualitative + descriptive)	2	5.4
Clinical validation	1	2.7
Randomized clinical trial	1	2.7
Pilot Randomized Clinical Trial	1	2.7
Year of publication		
2014	9	24.3
2015	7	18.9
2016	4	10.8
2017	8	21.6
2018	9	24.3

* n = Number of articles;

† Percentage of articles

The transition of care components presented in the studies are extremely varied, ranging from factors related to the patient^(^ 11 ^,^ 19 ^-^ 21 ^)^, going through the practices, strategies and tools used for the organization and execution of the transfer process^(^ 22 ^-^ 24 ^)^, to factors related to the unit to which the patient will be transferred^(^ 11 ^,^ 24 ^)^.

The factors related to the patient, identified in the primary studies, are severity of the disease^(^ 11 ^,^ 19 ^-^ 21 ^)^, presence of comorbidities^(^ 11 ^,^ 19 ^)^, presence of tracheostomy, older age^(^ 19 ^-^ 20 ^)^, altered state of consciousness, need for greater use of supportive therapies in the ICU, longer ICU stay, need for dialysis, and clinical causes of admission^(^ 20 ^)^. They are evidenced as predisposing factors for worse outcomes of the patients after being discharged from the ICU, such as adverse events or readmissions, in the perception of the professionals involved^(^ 11 ^,^ 21 ^)^, the association with the increase in readmission and mortality rates also being quantitatively verified^(^ 19 ^-^ 20 ^)^.

Some barriers are found for the continuity of care in the follow-up of the ICU, among these, the absence of specific discharge criteria and a feedback culture, the overestimation by the ICU team on the ability of the IU to monitor complex patients^(^ 25 ^)^, the change of health professionals, the changes in routines, and the substantial decrease in human resources and monitoring materials^(^ 13 ^,^ 21 ^,^ 24 ^-^ 25 ^)^.

On the other hand, several practices are identified as potential tools to improve the quality of transition of care and patient safety. One of the practices suggested is the adoption of a transfer checklist with items to check whether the patient is ready and the necessary adjustments before discharge, such as removal of invasive devices and medication reconciliation^(^ 23 ^,^ 26 ^)^. The oral or written communication was analyzed by several studies in different aspects. The use of a structured communication process using transfer of patients at bedside and standardized tools with multi-modal communication are strategies suggested^(^ 23 ^,^ 27 ^)^.

The involvement and preparation of the family is presented as an essential stage in the discharge process, with individual assessment of the information needs, preparing the family to adjust to a different environment with less staff, technology and support^(^ 28 ^)^. A study that investigated family members’ perceptions about the quality of care during the transfer process showed that the information about the transfer was significant for them, as they wanted to be part of the patient care and felt important when they had some vision and control over the necessary assistance. However, more than 20% felt that the information provided to them was inadequate^(^ 29 ^)^.

Another positive practice evidenced in the studies is being monitored or advised after discharge by members of the intensive care team. A qualitative study analyzing the perceptions of IU and ICU nurses on the benefits and challenges of the follow-up services of a post-intensive care group^(^ 30 ^)^ identified favorable points for both, such as the provision of additional care to the most vulnerable patients and continuity of intensive care, through periodic visits by the ICU team, in addition to the exchange of knowledge between the groups of nurses, in which the IU team’s unpreparedness for more complex care was often identified. The impact of post-discharge follow-up programs was also quantitatively assessed, showing a decrease in hospital stay and in the ICU readmission rate^(^ 31 ^)^.

Figure 3 illustrates the synthesis of the main practices, strategies and tools presented in the studies, with a potential positive or negative impact on the quality of the transition of care and the patients’ outcomes.

**Figure 3:** Practices, strategies and tools with potential positive or negative impact on the transition from the ICU to the IU. Porto Alegre, RS, Brazil, 2019

Practices, strategies and tools with potential positive or negative impact on the transition from the Intensive Care Unit to the Inpatient Unit	Impact
**Factors related to the Intensive Care Unit**	
Discharge at night^(11,21,24,32-35)^, at shift changes^(24)^ and on weekends^(19,21)^	Negative
Inadequate and/or non-standard communication of key information^(21,23-25,27,36-38)^	Negative
Premature discharge^(11,20,28,37-38)^	Negative
No discharge criteria^(11,14,21,25,37)^	Negative
Delays in the transfer^(23-24,39-40)^	Negative
Undefined care goals^(21,23-24,37)^	Negative
Incorrect destination after discharge^(11,22,38)^	Negative
Inadequate environment for an efficient communication^(21,23)^	Negative
Oral^(14,22,27,41-44)^ and written^(14,22,24,27-28,38,41,43,45)^ communication with the inpatient unit staff	Positive
Being monitored/advised by Intensive Care Unit professionals after discharge^(13-14,24-25,27,30-31,44,46-47)^	Positive
Discharge planning and guidelines for patients/family^(22-24,28-29,42,44-45,48)^	Positive
Medication reconciliation/review by the pharmacist^(14,22,24-26,49)^	Positive
Checklist/Transfer tools/Discharge protocols^(22-23,25-27,41)^	Positive
Anticipated discharge planning^(14,25,29,44)^	Positive
Participation of the patient and the family during the transfer^(22,24,41-42)^	Positive
Use of risk stratification tools/scores^(23,43,50)^	Positive
Transfer of care at the bedside^(23-24,27)^	Positive
Optimizing vital signs before discharge and reducing the need for intensive care^(29,37)^	Positive
Discharge to intermediate care units^(14,50)^	Positive
Institutional culture of valuing the transition of care process^(27)^	Positive
Transfer of care to their respective peers by all members of the multidisciplinary team^(43)^	Positive
**Factors related to the Inpatient Unit**	
Lack of qualification and experience by the staff ^(11,25,28,30,37-38,42)^	Negative
Reduced monitoring^(11,14,34,37)^	Negative
Reduced number of professionals^(11,25,27,37)^	Negative
Lack of available material resources^(21,25,27)^	Negative
Longer time until the first clinical evaluation of the patient^(14,24,43)^	Negative
Fragmentation of care in several teams^(11)^	Negative
Previous contact of the new team with the patient^(24,29)^	Positive
**Factors related to the Intensive Care Unit and the Inpatient Unit**	
Accountability for information sent and received^(23,29,43)^	Positive
Readmission risk alert^(43,50)^	Positive

The outcomes and adverse events analyzed are mostly focused on readmission^(^ 13 ^-^ 14 ^,^ 20 ^,^ 26 ^,^ 31 ^-^ 32 ^,^ 34 ^-^ 35 ^,^ 43 ^,^ 46 ^-^ 47 ^,^ 49 ^-^ 52 ^)^ and on mortality after discharge from the ICU^(^ 13 ^-^ 14 ^,^ 19 ^-^ 20 ^,^ 31 ^-^ 35 ^,^ 39 ^,^ 43 ^,^ 46 ^,^ 49 ^,^ 51 ^-^ 52 ^)^. The mortality rate after discharge from the ICU varies from 3.0%^(^ 46 ^)^to 30%^(^ 19 ^)^ according to the studies. Readmission affects 4.1%^(^ 51 ^)^to 9.2%^(^ 46 ^)^of the patients in any period of hospitalization, 2.9%^(^ 14 ^)^in 48 hours after the transfer and 2.7%^(^ 32 ^)^to 4.2%^(^ 13 ^)^ within 72 hours. Other clinical outcomes analyzed are length of hospital stay^(^ 31 ^-^ 32 ^,^ 35 ^,^ 39 ^-^ 40 ^,^ 49 ^)^, care provided by a Rapid Response Team (RRT)^(^ 26 ^,^ 43 ^)^, cardiac arrest^(^ 47 ^)^, and medications-related problems^(^ 49 ^)^. Outcomes such as anxiety, stress, and satisfaction of patients and families also appear in the studies^(^ 24 ^,^ 28 ^,^ 30 ^,^ 44 ^,^ 48 ^)^.

In studies with qualitative approaches on readmissions^(^ 21 ^)^ or post-discharge adverse events^(^ 11 ^)^, in the view of the care providers, factors related to the patient are listed, such as severity of the disease, undefined care goals, transfers at shift changes, nights or weekends, inadequate decision for discharge, professionals’ lack of experience^(^ 11 ^,^ 21 ^)^, limited resources, lack of institutional policies^(^ 21 ^)^, staff sizing and inadequate monitoring in the IU, choosing the wrong destination for the patient and fragmenting care in several teams^(^ 11 ^)^. Suboptimal communication among team members, an inappropriate environment and atmosphere for efficient communication and the lack of communication of key information are also elements identified as possible causes of readmissions^(^ 21 ^)^.

The association between the transition of care practices and the patients’ outcomes has varied results when assessed in quantitative studies. Some studies verify the association of certain practices with readmission and mortality rates, such as discharges at nights or weekends^(^ 19 ^,^ 32 ^,^ 34 ^-^ 35 ^)^. Discharges with delays of more than 24 hours showed a significant association with a higher incidence of*delirium* ^(^ 40 ^)^. Medication reconciliation or pharmaceutical intervention by reviewing medications prior to patient transfer may contribute to a decrease in the number and severity of medication-related problems; however, the impact on the mortality rate, length of hospital stay or ICU readmission is still inconclusive^(^ 49 ^)^. Using a medical alert form for the most vulnerable patients with guidance to the IU team, in addition to improvements in oral communication, tended to reduce readmission rates and calls to the RRT^(^ 43 ^)^.

On the other hand, some studies that tried to evidence the efficiency of strategies (such as the adoption of ICU discharge criteria, anticipated discharge planning, availability of intermediate care units, medication reconciliation, oral and written communication about the transfer, optimization of patient monitoring post-ICU and instructions to IU nurses) did not achieve significant results in reducing bad outcomes such as readmission and mortality^(^ 13 ^-^ 14 ^,^ 39 ^-^ 40 ^)^.

## Discussion

The 37 studies were published uniformly over time, showing a demand for knowledge in the last five years. More than 50% was conducted in the United States, Canada, Australia and New Zealand, and the other studies were concentrated in Europe, with only two carried out in Asia and three in Latin America (one in Brazil), which shows that the production of knowledge on the subject is concentrated in a few countries, possibly because it is a recent topic in the literature, which suggests the need for expansion and universalization to other regions that may present quite different aspects regarding the practices. A scoping review made in 2015^(^ 4 ^)^ also showed a concentration of most publications in the United States, Europe, and Australia. No other review was found in this format, which evaluates only adult patients.

According to the results identified, the transition of care in the discharge from intensive care is influenced by numerous components, whether intrinsic to the patient or related to the policies, practices or structure of the scenarios and professionals involved. Likewise, a previous study^(^ 4 ^)^ identified countless themes and factors related to professionals and the institution, which can act as facilitators or barriers to high quality care, confirming that being discharged from the ICU is a multifaceted and complex process.

With regard to factors related to the patient, conditions were identified that may predispose to worse outcomes after discharge from the ICU, especially conditions prior to discharge, such as the comorbidities and severity at the moment of hospitalization^(^ 11 ^,^ 19 ^-^ 21 ^)^, older age^(^ 19 ^-^ 20 ^)^, altered state of consciousness, and greater need for supportive therapies^(^ 20 ^)^. These findings are similar to other studies which identified that sicker patients, with greater severity at the moment of hospitalization^(^ 53 ^)^ and older patients^(^ 9 ^)^ had a greater chance of adverse events, readmission, and death after being discharged from intensive care. The altered state of consciousness was also found as a risk factor, along with polyneuropathy, myopathy and being discharged from the ICU using tube feeding, which often affect critically ill patients^(^ 53 ^-^ 54 ^)^.

Such factors are intrinsic to the patients, that is, they cannot be changed; therefore, they suggest the need to adopt specific strategies according to the profile and the individual demands of each patient, providing optimization and careful evaluation of the right moment for discharge, anticipated planning, more supervision for patients with greater severity, choice of the best destination unit or resizing of personnel and care for the most complex and dependent patients in the IU, in addition to stimulating greater family support, among other actions^(^ 11 ^,^ 23 ^)^.

Based on the 37 studies included, 30 practices, strategies and tools with a potential positive or negative impact were verified in the transition from the ICU to the IU, of which 21 were related to the ICU, seven were related to IU and two related to both. It is observed that most applies to the execution of the transfer, monitoring and care provided after the transfer, with a smaller portion representing actions taken in advance, such as planning individual needs, assessing readiness for discharge and preparing the people involved (team-patient-family).

A previous study^(^ 4 ^)^ also identified 30 factors related to the patient, professional or institution that can act as facilitators and barriers to high-quality care during discharge from the ICU. The main themes identified were patient’s and family’s needs and experiences, availability of complete and accurate information about the discharge, education related to the discharge for patients and families, discharge planning, standardization of the discharge process and the results of the patient discharge, including adverse events, readmission to the ICU and death. Few articles focused on education for health professionals working at the destination unit, medication reconciliation, and patient autonomy. The results were categorized into four different phases of the discharge process, namely: assessment of the patient’s readiness for discharge, discharge planning, discharge execution, and post-discharge follow-up^(^ 4 ^)^. Attention is drawn to studies that highlight the phase of discharge execution more than the other phases. In addition, actions focused on patient-centered care were commonly verified in studies with pediatric and neonatal patients, suggesting that there is a long way to go in the care of adult patients^(^ 4 ^)^.

It is noted that approximately half of the practices and strategies indicated by the studies can negatively influence the quality of the transitions. Among the main barriers are ineffective communication^(^ 21 ^,^ 23 ^-^ 25 ^,^ 27 ^,^ 36 ^-^ 38 ^)^, lack of planning, and choosing the inappropriate time for discharge, both from the point of view of time/day of discharge and the patient’s readiness to be discharged^(^ 11 ^,^ 14 ^,^ 19 ^-^ 22 ^,^ 24 ^-^ 25 ^,^ 27 ^-^ 28 ^,^ 30 ^,^ 32 ^-^ 35 ^,^ 37 ^-^ 38 ^,^ 42 ^)^. The ideal conditions for patient discharge should consider, in addition to the clinical aspects, their level of dependence, the availability of family support, and the capacity of the destination unit/team to meet their demands^(^ 28 ^,^ 37 ^)^.

The choice of the inappropriate time for discharge is sometimes driven by pressure due to the demand for ICU beds or the lack of responsibility for continuity of care, in which professionals fragment the process and do not feel responsible for the patients’ evolution after their transfer^(^ 28 ^,^ 38 ^)^. Survival after a critical illness is often associated with a long path and a potentially complicated recovery, which affects the quality of life of patients and their caregivers, and can persist for years after hospitalization^(^ 17 ^)^. On the other hand, delaying discharge from the ICU also brings unfavorable outcomes, such as inefficient use of hospital resources and delay in the hospitalization of other critical patients^(^ 8 ^,^ 11 ^)^.

The decision to discharge from the ICU cannot be taken separately and in a single moment, it must be discussed throughout the hospitalization to enable a better assessment of the best moment, the planning of the practices and strategies that best apply to each case, anticipating demands for physical and human resources and for preparation of the patient and family for an adequate continuity of care in the transition^(^ 25 ^,^ 29 ^)^. Therefore, the assessment for discharge must integrate daily discussions and use minimum criteria for a safe transfer, if possible, integrating risk stratification strategies to alert those involved and think about specific actions to prevent unfavorable outcomes.

Some studies suggest the use of scores to define patient readiness for discharge, such as the Stability and Workload Index for Transfer (SWIFT)^(^ 50 ^)^scale, which includes in the score the patient’s original unit and length of stay in the ICU, the Glasgow Coma Scale (GCS), the ratio of partial arterial oxygen pressure (PaO2)/inspired fraction of oxygen (IfO2) and arterial pressure of carbon dioxide (ApCO2). Other studies using risk scores to guide the decision to discharge^(^ 8 ^,^ 55 ^)^ include physiological, clinical and laboratory results measures like vital signs, clinical assessment, Braden scale scores, laboratory tests, and heart rate, jointly. Altered vital signs and level of consciousness at the moment of discharge are also suggested as predictors of risk for clinical deterioration in the IU independently^(^ 10 ^,^ 12 ^)^, or composing a scale^(^ 56 ^)^.

One of the main pillars for a quality transition of care is effective communication, as it permeates all moments and actors, so that several positive or negative aspects related may include oral and written communication, such as, for example, discharge summary and/or information transfer forms to the next caregiver. Several studies^(^ 21 ^,^ 23 ^-^ 25 ^,^ 27 ^,^ 36 ^-^ 38 ^)^ revealed inadequate communication of key information and lack of standardization. Ineffective communication can be caused by many factors, such as different expectations between those who pass on and those who receive the information, cultural issues (absence of teamwork and lack of respect among professionals), inadequate time for this activity and lack of methods or standardized tools^(^ 57 ^)^.

Similarly to the findings of this review, in which strategies are suggested for improving communication, such as the use of standardized tools, face-to-face interaction with the professional to whom the patient is transferred, use of checklists, identification of the best time and place to transfer and inclusion of the patient and family^(^ 23 ^-^ 24 ^,^ 27 ^)^, other studies confirm that the use of standardized tools, adequate environment and time, eye contact, and active listening are crucial factors in ensuring effective communication^(^ 57 ^-^ 58 ^)^.

The preparation and discharge guidelines for patients and their families were mentioned in several studies as a fundamental stage in the process^(^ 22 ^-^ 24 ^,^ 28 ^-^ 29 ^,^ 42 ^,^ 44 ^-^ 45 ^,^ 48 ^)^; however, the best strategy is not yet established. Patients and family members are still often excluded from the transition of care process and the information provided is sometimes conflicting, diverging between professionals or teams, and unclear instructions on future care are offered, with technical terms and little time dedicated to this activity^(^ 58 ^-^ 60 ^)^. The discharge planning must start with the information of the plan to the patient and the family members, allowing for the activation of support systems that may be necessary and guiding them on the care received, the planned care, the discharge process, and how the destination unit works^(^ 4 ^)^.

The medication review by a pharmacist before transferring the patient to the IU proved to be effective in reducing the number and severity of medications-related problems^(^ 49 ^)^, although the impact on outcomes such as mortality and readmission is inconclusive^(^ 14 ^,^ 49 ^)^. A recent study^(^ 61 ^)^ found that medication reconciliation by a pharmacist reduced errors in medication transfer, potential adverse events, and related costs.

The choice of the destination can be a decisive factor in the patient’s outcomes, as in the example of the availability of intermediate care units; however, its impact is still controversial, both in the analyzed articles^(^ 14 ^,^ 50 ^)^, and also as noted by other authors^(^ 52 ^,^ 62 ^)^. In a study conducted in Brazil, referral to an intermediate care unit did not affect in-hospital mortality or the incidence of readmissions in the ICU^(^ 52 ^)^, while other study showed a significantly lower risk of readmission for patients transferred to an intermediate care unit^(^ 62 ^)^.

The follow-up or guidance after discharge by members of the intensive care team is one of the strategies with a potential positive impact on the patients’ outcomes, as evidenced by some studies, showing a decrease in the length of hospital stay^(^ 31 ^)^ and in the ICU readmission rate^(^ 31 ^,^ 47 ^)^; however, there was no consensus^(^ 13 ^-^ 14 ^)^. Corroborating these findings, a meta-analysis carried out in 2014 identified that transition of care programs focused on the follow-up after discharge from the intensive care were associated with reduced risk of readmission to the ICU^(^ 6 ^)^. The programs were developed by medical emergency teams or liaison nurses who did follow-up or offered consultations to patients after discharge from the ICU, but the team members did not always have prior contact with the patient before discharge. Thus, there is a need for more research to prove the real impact of the programs and services of follow-up after discharge from the ICU.

The readmission^(^ 13 ^-^ 14 ^,^ 20 ^,^ 26 ^,^ 31 ^-^ 32 ^,^ 34 ^-^ 35 ^,^ 43 ^,^ 46 ^-^ 47 ^,^ 49 ^-^ 52 ^)^ and death^(^ 13 ^-^ 14 ^,^ 19 ^-^ 20 ^,^ 31 ^-^ 35 ^,^ 39 ^,^ 43 ^,^ 46 ^,^ 49 ^,^ 51 ^-^ 52 ^)^ outcomes were more analyzed in the studies than other outcomes, with mortality rates showing a wide variation (3-30%)^(^ 19 ^,^ 46 ^)^. Few studies were devoted to assessing other adverse outcomes; however, it is important to note that not all patients undergoing an inadequate transition process evolve to death or readmission but, even so, they may be subjected to unwanted repercussions with serious consequences, such as the need to change or increase the length of treatment, increased length of hospital stay, disabilities, increased hospital costs, and dissatisfaction^(^ 9 ^)^.

A recent study found that 21% of the discharged patients had post-ICU deterioration, including cardiac arrest, RRT calls, and readmission^(^ 63 ^)^. Patients undergoing lung transplantation and other thoracic surgery, as well as advanced age, increased severity of the disease estimated by the Acute Physiology and Chronic Health Evaluation III (APACHE III) score, bradycardia, abnormal levels of albumin in the admission to the ICU, hyperkalemia and high level of activated partial thromboplastin time (APTT) at discharge from the ICU, presented a higher risk regardless of deterioration. In addition to these factors intrinsic to the patient, it was found that the patient being ready for discharge less than 48 hours before was an independent risk factor, which may indicate insufficient time for planning the transition of care^(^ 63 ^)^. A broader analysis of the adverse outcomes due to failures in the ICU discharge process is essential, considering its potential impact on outcomes that have an impact on the quality of life of the patients and their families.

The limitations of this scoping review include the fact that the authors delimited the published primary studies, that is, review studies and gray literature were not included, and that there were language restrictions. The heterogeneity of the studies analyzed, both in terms of methodology and diversity of outcomes and presentation, limits the comparison between the data. In addition, it is possible that a precise and exhaustive data extraction was not achieved, given the number and plurality of outcomes included, although it was performed by two reviewers, using a tool to systematically conduct data extraction. The classification of the impact of the practices, strategies or tools as positive or negative, although made independently by both reviewers with subsequent consensus, is relatively subjective since many studies do not report the outcomes clearly, hindering their interpretation.

A series of practices, strategies and tools were indicated to have a potential to assist in the coordination of the discharge process, improving the sometimes unfavorable outcomes of critical patients with complex care needs, even after leaving the intensive care environment. Such results reinforce the complexity of the ICU discharge process, in which many factors are involved and indicate critical points that can be improved in the transition of care, suggesting that the adoption of fragmented strategies, involving only a few phases, is likely to be unsuccessful.

In spite of that, the results of this review indicate that there is no consensus regarding the factors that influence transition of care after discharge from the ICU, the best practices or strategies that can be effective, or even regarding the repercussions and outcomes caused to the patient-family, which shows a wide range of themes to be explored in search of better scientific evidence on the subject. New studies should discuss the best strategies but should not be limited to a single practice, strategy or tool, as it is a complex process that needs to cover the several components and characters involved.

## Conclusion

This review made it possible to identify components and to map the transition of care practices used in the discharge of adult patients from the ICU to the IU. Thirty practices, strategies and tools were used to organize and execute the transfer process. Some of the factors that stand out are related to the ICU and the hospitalization unit to which the patient was transferred, and cross-sectional to the units, to the teams involved and the institution itself, which may be associated with positive or negative outcomes. In addition, factors intrinsic to the patient, such as comorbidities and severity of the disease at the time of hospitalization, were associated with worse outcomes after discharge from the ICU.

Practices such as discharge at night or on weekends showed association with increased rates of readmission and mortality. Medication reviews by pharmacists and the adoption of warning systems for patients at risk in the IU showed a tendency to reduce adverse outcomes, such as drug-related problems, RRT calls and readmission. Other practices are recognized as potential predictors or protectors for outcomes after discharge from the ICU; however, there was no consensus in the literature.

Therefore, the association between transition of care and the outcome of the patient after transfer to the IU is still inconclusive, further research studies being necessary to assess the impact of different practices, strategies, and tools. There are also new research opportunities to evaluate the implementation of such practices, isolated and combined, in different scenarios of clinical practice, seeking to identify the effect on the quality of the intensive care discharge process.

In addition to the relevance in the field of research, this study offers contributions to professionals, patients and families, showing the need for a broader transition of care process, with reformulation of practices, considering the complexity involved since the patient’s hospitalization in the ICU until the stabilization in the IU, for the quality of continuous care. The adoption of transition of care programs can be an effective management tool for health institutions, reducing the length of hospital stay and improving the use of resources.
